# Clinical Asthma Phenotypes and Therapeutic Responses

**DOI:** 10.1155/2013/824781

**Published:** 2013-03-31

**Authors:** M. Zedan, G. Attia, M. M. Zedan, A. Osman, N. Abo-Elkheir, N. Maysara, T. Barakat, N. Gamil

**Affiliations:** ^1^Allergy, Clinical Immunology and Respiratory Medicine Unit, Faculty of Medicine, Mansoura University, P.O. 35516 Box 50, Mansoura, Egypt; ^2^Pediatric Department, Faculty of Medicine, Mansoura University, P.O. 35516, Mansoura, Egypt; ^3^Clinical Pathology Department, Faculty of Medicine, Mansoura University, P.O. 35516, Mansoura, Egypt; ^4^Pharmacology Department, Faculty of Pharmacy, Mansoura University, P.O. 35516, Mansoura, Egypt

## Abstract

Asthma is a heterogeneous disease that means not all asthmatics respond to the same treatment. We hypothesize an approach to characterize asthma phenotypes based on symptomatology (shortness of breath (SOB), cough, and wheezy phenotypes) in correlation with airway inflammatory biomarkers and FEV1. We aimed to detect whether those clinical phenotypes have an impact on the response to asthma medications. Two hundred three asthmatic children were allocated randomly to receive either montelukast (5 mg at bed time) or fluticasone propionate (100 ug twice daily) for 8 consecutive weeks. Serum concentrations of IL-2Rs, ICAM-1, VCAM-1, total IgE, eosinophilic %, eosinophil cationic protein (ECP), and FEV1 were done before and after treatment to patients and once to controls. Children who have SOB were found to have higher levels of total sIgE, older age, and longer disease duration, and they responded to fluticasone alone. Cough group was found to have higher levels of eosinophilic % and sECP, younger age, shorter disease duration and responded to montelukast alone. Wheezy group showed mixed pattern and responded to both medications. *Conclusion.* Although there is variability in response to ICS and LTRAs, we did identify characteristics of patient that should guide the clinician in the choice of asthma medications.

## 1. Introduction

 Evidence is increasing that asthma is a heterogeneous disease constituted by overlapping separate syndromes with probably different, but yet undefined, causes and natural histories. There is a need to identify each of these groups of patients (the so-called asthma phenotypes), whose clinical and prognostic characteristics and responses to treatment may be heterogeneous between groups and homogeneous within each group [1].

 All asthmatics, by definition, share a common physiologic abnormalities of reversible airflow obstruction detected by spirometry, airway hyperreactivity, and symptoms that can include shortness of breath, wheezes, and cough. Despite these shared features, a great heterogeneity was noticed in the severity of airway obstruction, clinical phenotypes, degree of reversibility, and the amount of improvement in response to asthma medicines [2]. These phenotypes include allergic and nonallergic asthma. Other phenotypes defined by clinical or physiological categories (i.e., severity, age at onset, and chronic airway obstruction), by asthma triggers (i.e., viral, exercise, occupational allergens, or irritants), by their pathobiology (i.e., eosinophilic, neutrophilic, and paucigranulocytic asthma), or by their course (i.e., early transient/persistent/late onset wheeze) have also been proposed [3–6]. 

 In an attempt to understand the mechanisms for these variable clinical phenotypes and response to medications, many approaches have been taken to assign asthmatics to distinct phenotypes that can predict disease course and treatment response [2]. Thus, identification of asthma phenotypes should also lead to increasing the understanding of underlying pathobiology that contributes to a particular phenotype [3]. The huge advances in asthma pathology achieved in the last decades have resulted in discussions about whether asthma definition and classification should be revised, but their implications in the clinical practice are still missing [1].

 We hypothesize an approach to classify asthma phenotypes based on symptomatology in correlation with cytokine profile and airway inflammatory biomarkers as a trial to individualize asthma treatment. Beside our trial to characterize the proposed clinical asthma phenotypes, we aim to detect whether those clinical asthma phenotypes may affect the response to the main controller medications. This trial may translate the results into a simple clinical guide that can help to tailor the asthma medicines.

## 2. Subjects and Methods

 Patients (*n* = 203) 8 to 14 years of age with partially controlled asthma were enrolled in the study after validation of their symptoms [7, 8] into shortness of breath, cough, and wheezy phenotype groups. They had asthma symptoms or rescue medication use on average of 3 or more days per week during the previous 4 weeks and improvement in FEV1 ≥ 12% after maximal bronchodilation. They had no corticosteroid treatment within 4 weeks, no antihistamines within 3 months, no montelukast treatment within 2 weeks, and no history of respiratory tract infection within 4 weeks of enrollment. Children were excluded for the following comorbidities such as chronic cardiopulmonary disease, concurrent pneumonia, nasal polyps, obesity, and gastroesophageal reflux. Also, patients under immunotherapy were excluded. The study included 44 healthy controls (mean age 10.20 ± 0.22 years) of matched age and sex without apparent evidence of allergic diseases. We defined the level of asthma control according to established guidelines of Global Strategy for Asthma Management and Prevention [9]. Informed consent was obtained from all parents of patients and healthy controls and approved by Ethical Committee of Mansoura Faculty of Medicine, Egypt. 

### 2.1. Study Design

 According to the clinical phenotypes based on validated symptomatology [7, 8], asthmatic children were divided into 68 children presented solely with shortness of breath (defined by the patient as chest tightness, labored breathing, and difficulty in drawing sufficient breath, heavy breath) with a mean age of 9.7 ± 3.2 years, 63 children presented solely with cough without other symptoms such as dyspnea or wheezes [10] with a mean age of 9.8 ± 2.3 years, and 72 children presented predominately with wheezes (defined by the patient as creaking, rattle, whistling, and jingling) with a mean age of 9.3 ± 1.2 years. In either group of patients, the presenting clinical phenotype had to be persistent during their followup; those who had variable clinical presentation between the phenotypes were excluded.

After a 10- to 14-day characterization period, participants were randomized to either line of treatment using an active ICS, inhaled fluticasone propionate 100 *μ*g twice daily, for 8 consecutive weeks (Flixotide Diskus, Glaxo Wellcome Egypt, under license from Glaxo Wellcome Operations, UK), or montelukast 5 mg chewable tablet once daily at bedtime, for 8 consecutive weeks (Singulair, Merck, Whitehouse Station, NJ, USA). Short-acting *β*2 agonists (ventolin, Glaxo Wellcome, London, UK) were administered as a rescue medication. During treatment, patients were asked to regularly visit the outpatient clinic on weekly bases to evaluate compliance to therapy and degree of asthma control. 

 Evaluation was done using peak flow measurements (AM1, Jaeger-Toennies GmbH, Hoechberg, Germany) and asthma symptom scores. An asthma-free day was defined as a day without the following: daytime or nighttime symptoms, use of rescue salbutamol for asthma symptoms or low peak flow, asthma health care use, or missed school or work for asthma symptoms.

 Patients were excluded from the study if they showed deterioration of their clinical status accidentally used other controller medication, and were willing to get back to their regular treatment.

 Serum concentrations of soluble intercellular adhesion molecule (sICAM), soluble vascular cell adhesion molecule (sVCAM), soluble interleukin-2 receptor (sIL-2R), total serum immunoglobulin E (sIgE), peripheral eosinophil %, serum eosinophilic cationic protein (sECP), and pulmonary function tests (PFTs) were done before and after treatment for patients and were done once to controls.

### 2.2. Laboratory Tests

Blood samples were obtained from each patient and healthy controls. Blood was collected into serum separate tubes in two aliquots. One aliquot was used for complete blood count for eosinophils and expressed in cell/mm. The other was kept for 30 min to clot and then centrifuged at 3,000 rpm for 10 min. Aliquots of serum were stored at −70°C before analysis. In each sample, sICAM-1, sVCAM-1, sIL-2R, and total IgE levels were measured by using immunoassay techniques. sICAM-1, sVCAM-1, and sIL-2R were measured by using ELISA KITS from DIACLONE Research, France. Serum IgE levels were measured by using IgE EISA KITS from Aptech Services. Serum Eosinophilic cationic protein (ECP) assay was done using IMMULITE system from DPC (Diagnostic Procedure Corporation) Los Angeles, CA, USA [11].

### 2.3. Pulmonary Function Tests (PFTs)

 FEV1 was measured by spirometry (Master Screen body); the highest reading of three successive measurements was taken. Reference values were computed according to the recommendations of the American Thoracic Society (ATS) standards of Acceptability and Reproducibility [12].

### 2.4. Statistical Methods

 The target sample size of 203 randomized participants provided 85% statistical power for detecting a significant correlation between the study medications. Statistical analysis was done by using SPSS (Statistical Package for Social Science) software (version 12.0, SPSS Inc., Chicago, IL, USA). The results were analyzed by using paired student's *t*-test and Wilcoxon rank test to assess differences in serum levels of ICAM-1, VCAM-1, IL-2R, ECP, and total IgE at the beginning and at the end of treatment. Mann-Whitney *U* test was used to compare both groups. Statistical significance was defined as *P* < 0.05.

## 3. Results

**Figure 1:**
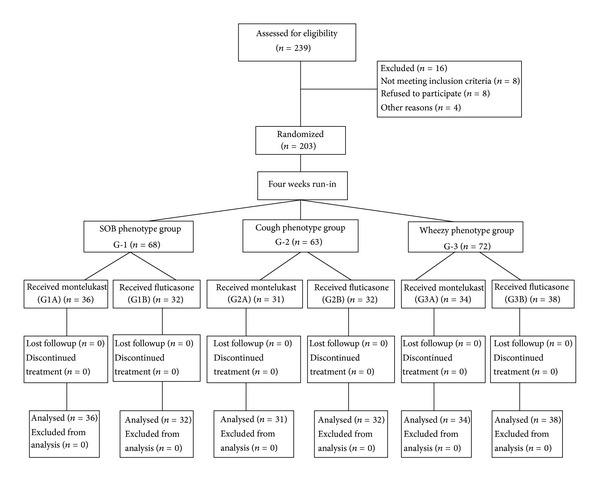
Flow diagram showing the numbers of children involved in each stage of the study.

Two hundred thirty-nine asthmatic children were enrolled in the study, 203 had successfully completed both treatment arms, and 15.06% of the participants did not complete the study (Figure 1). The three studied groups (SOB, cough, and wheezy) showed insignificant difference as regards age and sex. There was statistical significance between asthmatic children and healthy controls regarding FEV1%, serum levels of IgE, peripheral eosinophilic %, serum eosinophilic cationic protein (sECP), soluble intercellular adhesion molecule (sICAM-1), soluble vascular cell adhesion molecule (sVCAM-1), and soluble interleukin-2 receptor (sIL-2R) (Table 1).

 Before treatment, shortness of breath (SOB) group showed significant increase in total serum IgE when compared with both cough and wheezy groups. Whereas cough phenotype group showed significant increase in both peripheral eosinophilic % and sECP when compared with SOB and wheezy groups. On the other aspect, wheezy group showed mixed pattern in the form of significant increase in peripheral eosinophilic % and sECP when compared with SOB group, beside a significant increase in total serum IgE when compared with cough group (Table 1).

 There was agreement in the responses to the two medications at the end of 8-week treatment period, with clear difference in the response according to the clinical phenotype. Shortness of breath group responded to fluticasone alone with significant improvement in both FEV1 and asthma symptom scores as well as significant decrease of peripheral eosinophilic percentage, sECP, sICAM, and total serum IgE (Table 2). On the other aspect, cough group responded to montelukast alone with significant improvement in both FEV1 and asthma symptom scores and with significant decrease in peripheral eosinophilic % and sECP (Table 3). 

Wheezy group responded to both medications in which mean (SD) FEV1 percentage of improvement was 14.1% for fluticasone and 8.6% for montelukast. Also, the same groups showed significant improvement in asthma symptom scores with significant decrease in peripheral eosinophilic percentage and sECP in response to both medications. Overall, the difference in the response for the two medications in wheezy group was found to be significant with upper hand to fluticasone (Table 4).

Table 5 showed comparison between phenotypic clinical parameters as well as peripheral eosinophilic percentage among cases controlled with either montelukast or fluticasone. Cases controlled with montelukast were found to have cough phenotype with eosinophilic pattern, younger age (<10 years) with shorter disease duration (<10 years), and female gender with positive family history of asthma. On the other aspect, cases controlled with fluticasone were found to have shortness of breath phenotype, older age with longer disease duration, and male gender with negative family history of asthma.

## 4. Discussion

 Asthma is increasingly considered a syndrome, with diverse overlapping pathologies and phenotypes contributing to significant heterogeneity in clinical manifestations, disease progression, and treatment response [13]. Better defining asthma phenotypes may improve the understanding of the underlying pathobiology of the phenotypes and lead to targeted therapies for individual phenotypes [14].

 Our hypothesis is based on the clinical heterogeneity of asthma symptoms. We are exploring whether each clinical phenotype (SOB, cough, and wheeze) has its own specific features and inflammatory biomarkers aiming to characterize those clinical asthma phenotypes and to look for their implications on the response to the main controller asthma medicines. Our study described a wide variability between the proposed clinical phenotypes in which the SOB phenotype group were found to have elevated levels of total sIgE, older age (>10 years), male gender, and longer disease duration with negative family history of asthma. Whereas cough phenotype group were found to have an eosinophilic pattern, younger age (<10 years), female gender, and shorter disease duration, with positive family history of asthma. On the other aspect, a mixed IgE and eosinophilic pattern were noticed in the wheezy phenotype group.

 The children in our study were treated for eight consecutive weeks with two controller asthma medicines ICS and LTRA. The effect of each medicine was found to vary according to the proposed clinical asthma phenotype. SOB phenotype group responded to fluticasone alone by significant improvement in both FEV1% and asthma symptom scores and significant decrease of peripheral eosinophilic%, sECP, total sIgE, and sICAM-1. The response to ICS in SOB phenotype group may be explained by a proposed cytokine pathway in this phenotype or attenuated cysteinyl leukotriene pathway in this group; however, the detailed mechanisms remain to be clarified by future controlled studies. 

 On the other aspect, cough phenotype group responded to montelukast alone by significant improvement of both FEV1 and asthma symptom scores with significant decrease in peripheral eosinophilic% and sECP. Also in the current study, both of these classes of medicines fluticasone and montelukast were found to be effective in the wheezy group by significant improvement in both FEV1 and asthma symptom scores with significant decrease of peripheral eosinophilic % and sECP. The differential response between the two medications was found to be significant with upper hand to fluticasone. 

Overall, in current research, cough group was found to have an eosinophilic pattern and responded to montelukast alone which may be explained by an underlying leukotriene-driven eosinophilic inflammation [15], whereas SOB group was found to have higher levels of total sIgE and responded to ICS but not to LTRAs. On the other aspect, wheezy group that responded to both medications ICS and LTRAs was found to have a mixed eosinophilic and IgE mediated pattern and this may need further studies to delineate the underlying pathogenesis.

 Data emerging from the present study showed that the SOB phenotype group had significant increase in total sIgE levels with significant decrease in FEV1 in comparison with cough and wheezy groups. These findings may characterize and reflect the severity of this group on a clinical background. A number of previous studies indicated that total sIgE levels might reflect the severity of asthma. TENOR study showed that mean sIgE levels were significantly higher in children with severe asthma than in those with moderate or mild disease [16]. Naqvi, 2007, found that higher total sIgE among 739 African-American, Mexican, and Puerto Rican adults and children with asthma was associated with lower baseline lung function and more severe asthma [17]. Another cross-sectional cohort study of 157 asthmatic children, aged 5 to 15 years, reported a correlation between disease severity and specific IgE to house dust mite, *Dermatophagoides pteronyssinus* [18].

 Different clinical research has suggested an emerging clinical usefulness of eosinophilic percentage and serum eosinophil granule proteins in the assessment and management of asthma, of which ECP has been most widely characterized and researched. Eosinophils are a characteristic feature of the pathology of asthma [19] in which the granular constituents of eosinophils are cytotoxic and cause desquamation and destruction of bronchial epithelium [20], which may lead to bronchial hyperresponsiveness [21]; lipid mediators secreted from eosinophils, such as leukotrienes C4, D4, and E4 and platelet activating factor, can induce bronchoconstriction, vascular permeability, and bronchial hyper responsiveness [20]. Peripheral eosinophils and s-ECP levels were found to be sensitive markers for asthma severity [22] and assessment of asthma control [23, 24]. Therapy guided by eosinophilic % has proven to be effective with cutoffs generally less than 2% of forced sputum or bronchoalveolar lavage (BAL) [25]. Eosinophilic asthma patients tend to respond well to steroids and bronchodilators but have a high frequency of flares [26, 27]. 

 In conclusion, although there is a variability in response to ICS and LTRAs, we did identify the characteristics of patient that should guide the clinician in the choice of asthma controller medications. Children who have SOB as main complaint with high levels of total sIgE should receive ICS therapy, whereas cough phenotype group with high levels of eosinophilic % and sECP should receive montelukast. While wheezy group with mixed eosinophilic and IgE mediated pattern could receive therapeutic trials of either ICS or LTRAs with an assessment of the response.

## Figures and Tables

**Table 1:** Laboratory and demographic data of the studied groups.

	G1	G2	G3	G4	*P1 *	*P2 *	*P3 *	*P4 *	*P5 *	*P6 *
FEV1% predicted	66.3± 1.3	68.8± 1.2	67.2 ± 0.9	91.8± 1.1	<0.001*	0.8	<0.001*	<0.001*	<0.001*	<0.001*
Total sIgE (IU/mL)	219.1± 14.9	132.8± 30.3	183.8± 17.6	52.0± 12.59	<0.001*	<0.001*	<0.001*	<0.001*	<0.001*	<0.001*
Peripheral eosinophilic%	5.2± 0.69	8.13± 0.83	7.66± 1.08	1.7± 0.71	<0.001*	0.1	<0.001*	<0.001*	<0.001*	0.03
sECP (*µ*g/L)	36.21± 7.05	74.33± 12.38	49.7± 8.25	18.32± 5.19	<0.001*	<0.001*	<0.001*	<0.001*	<0.001*	<0.001*
sICAM-1 (ng/mL)	716± 56.4	711.5± 70.9	704± 62.2	402 ± 0.7	<0.001*	0.9	0.3	<0.001*	0.2	<0.001*
sVCAM-1 (ng/mL)	879.9± 269.1	847.2± 230.2	864± 239.2	550 ± 0.3	<0.001*	0.7	0.8	<0.001*	0.3	<0.001*
sIL-2R (pg/mL)	3922± 379.1	3453.8± 544.05	3433.7± 349.2	1980 ± 0.5	<0.001*	0.3	<0.001*	<0.001*	<0.001*	<0.001*
Age (y)	9.7± 2.3	9.8± 2.3	9.3± 1.2	10.2± 0.22	0.66	0.66	0.66	0.66	0.66	0.66
Male	25 (52.08%)	23 (53.4%)	27 (51.92%)	21 (47.72%)	0.6	0.6	0.6	0.6	0.6	0.6
Female	23 (47.9%)	20 (46.51%)	25 (48.07%)	23 (52.27%)	0.62	0.62	0.62	0.62	0.62	0.62
Duration of asthma (y)	5.4 ± 2.4	5.5 ± 3.4	5.3 ± 2.4			0.71	0.71		0.71	
FH: positive	28 (58.3%)	24 (55.81%)	29 (55.76%)	Negative						
FH: negative	20 (41.6%)	19 (44.18%)	23 (44.23%)	Negative						

Data are expressed as mean (SD); *P < 0.05 is considered significant.

IgE: immunoglobulin E, sECP: serum eosinophilic cationic protein, and FH: family history of asthma.

G1: shortness of breath group (SOB) group, G2: cough group, G3: Wheezy group, and G4: controls.

*P1* : G2 versus G4, *P2* : G2 versus G3, *P3* : G2 versus G1, *P4* : G3 versus G4 , P5: G3 versus G1, and *P6* : G1 versus G4.

**Table 2:** Effect of montelukast versus fluticasone on pulmonary function and airway inflammatory biomarkers in shortness of breath (SOB) group (G1).

	G1A			G1B			*P*
	Before treatment	After treatment	Percentage change	Before treatment	After treatment	Percentage change	
FEV1 % predicted	66.2 ± 0.9	69.3 ± 1.2	4.6%	65.9 ± 0.8	75.2 ± 2.1*	14.1%	<0.001
Total sIgE (IU/mL)	219 ± 12.81	201 ± 9.8	−8.22%	219.2 ± 17.2	161.8 ± 22.3*	−26.18%	<0.001
Peripheral eosinophilic%	4.8 ± 0.9	3.9 ± 0.2	−18.75%	5.2 ± 0.1	2.1 ± 0.01*	−59.61%	<0.001
sECP (*μ*g/L)	37.9 ± 6.2	32.9 ± 4.1	−13.19%	35.1 ± 8.2	18.3 ± 6.2*	−47.86%	<0.001
sICAM-1 (ng/mL)	712.2 ± 44.9	701 ± 22.3	−1.57%	719 ± 33.8	633.9 ± 72.2*	−11.83%	<0.001
sVCAM-1 (ng/mL)	882 ± 240.9	870 ± 198.8	−1.36%	878.3 ± 203.2	869.8 ± 188.3	−0.97%	0.88
sIL-2R (pg/mL)	3838 ± 374.5	3810 ± 213.9	−0.73%	3896 ± 361.2	3860 ± 290.3	−0.92%	0.78

G1A: SOB group treated with montelukast. G1B: SOB group treated with fluticasone.

Data are expressed as mean (SD). *P < 0.05 is significant (for each group before and after treatment).

Mann-Whitney *U*-test was used to compare both groups.

**Table 3:** Effect of montelukast versus fluticasone on pulmonary function and airway inflammatory biomarkers in cough phenotype group (G2).

	G2A			G2B			*P*
	Before treatment	After treatment	Percentage change	Before treatment	After treatment	Percentage change
FEV1 % predicted	66.3 ± 2.1	79.2 ± 2.2*	19.46%	67.9 ± 2.4	69.2 ± 0.8	1.9%	0.6
Total sIgE (IU/mL)	134.2 ± 32.1	130.1 ± 29.7	−3.05%	130.6 ± 28.2	125.8 ± 20.9	−3.67%	0.82
Peripheral eosinophilic%	8.31 ± 0.29	4.3 ± 0.1*	−48.2%	7.6 ± 0.71	6.2 ± 0.3	−18.4%	0.52
sECP (*μ*g/L)	76.22 ± 9.8	44.9 ± 3.2*	−41.09%	72.9 ± 1.2	68.3 ± 2.3	−6.3%	0.56
sICAM-1 (ng/mL)	713.2 ± 69.2	698.2 ± 53.2	−2.1%	709.8 ± 71.2	698.2 ± 66.2	−1.63%	0.67
sVCAM-1 (ng/mL)	850.3 ± 219.8	832.2 ± 188.9	−2.13%	844.3 ± 211.2	813.2 ± 189.3	−3.68%	0.78
sIL-2R (pg/mL)	3461 ± 488.2	3319 ± 378.2	−4.1%	3453.1 ± 399.2	3402.2 ± 298.8	−1.47%	0.46

G2A: cough group treated with montelukast. G2B: cough group treated with fluticasone.

Data are expressed as mean (SD). *P < 0.05 is significant (for each group before and after treatment).

Mann-Whitney *U*-test was used to compare both groups.

**Table 4:** Effect of montelukast versus fluticasone on pulmonary function and airway inflammatory biomarkers in wheezy phenotype group (G3).

	G3A			G3B			*P*
	Before treatment	After treatment	Percentage change	Before treatment	After treatment	Percentage change	
FEV1% predicted	69.2 ± 1.8	75.2 ± 2.1*	8.6%	70.2 ± 1.1	80.1 ± 0.9*	14.1%	<0.001
Total sIgE (IU/mL)	182.2 ± 13.2	175.1 ± 11.2	3.89%	184.8 ± 11.8	165.8 ± 9.8	10.28%	0.9
Peripheral eosinophilic%	7.4 ± 0.9	4.2 ± 0.19*	−43.24%	6.9 ± 0.8	2.8 ± 0.2*	−59.42%	<0.001
sECP (*μ*g/L)	50.2 ± 7.1	32.3 ± 6.2*	−35.65%	47.8 ± 7.1	22.9 ± 4.3*	−52.09%	<0.001
sICAM-1 (ng/mL)	701.9 ± 59.2	692 ± 63.2	1.41%	708 ± 60.1	680 ± 50.2	3.95%	0.8
sVCAM-1 (ng/mL)	860.2 ± 219.8	823 ± 198.2	4.32%	866.9 ± 230.9	811 ± 180.9	6.44%	0.78
sIL-2R (pg/mL)	3428 ± 329.2	3411 ± 230.1	0.49%	3439 ± 330.2	3400 ± 310.1	1.13%	0.86

G3A: wheezy group treated with montelukast. G3B: wheezy group treated with fluticasone.

Data are expressed as mean (SD). *P < 0.05 is significant (for each group before and after treatment).

Mann-Whitney *U*-test was used to compare both groups.

**Table 5:** Demographic data of patients that achieved control using treatment with either montelukast or fluticasone.

Studied parameters	Controlled Cases Montelukast (%)	Controlled Cases Fluticasone (%)	*P*
Age			
<10 Years	88.8	30.3	0.006*
>10 Years	11.2	69.7	
Sex			
Male	13.3	41.6	0.005*
Female	86.7	58.4	
Asthma-free days per week	7	7	
Asthma phenotypes			
Cough phenotype	86	20	0.005*
SOB phenotype	15	80	
Family history of asthma			
Positive	88.8	25	0.006*
Negative	11.2	75	
Body mass index (kg/m^2^)			
<20%	100	75	0.333
20–25%	0	8.4	
>25%	0	16.6	
Duration			
<5 Years	55.5	25	
5–10 Years	33.3	58.4	0.006*
>10 Years	11.2	16.6	
Eosinophilic% before treatment			
Mean + SD	8.0 + 2.0	7 + 2.9	0.19
Median	8.0	6.0	

*P significant <0.05.
